# Kidney transplant in Nigeria: a single centre experience

**DOI:** 10.11604/pamj.2016.25.112.7930

**Published:** 2016-10-25

**Authors:** Umezurike Hughes Okafor

**Affiliations:** 1 Renal unit, Department of Medicine, ESUT Teaching Hospital Parklane, Enugu, Nigeria

**Keywords:** Chronic kidney disease, kidney transplant, immunosuppression, outcome, survival

## Abstract

**Introduction:**

Kidney transplant is the preferred renal replacement therapy for patients with end stage kidney disease. However management of patients with kidney transplant in resource poor countries is evolving and groaning under several mental, financial and infrastructural challenges. The objective of the study is to evaluate the management of patients with kidney transplant in a kidney care Centre in Nigeria.

**Methods:**

This was a non-randomized prospective study. The study population were post-transplant patients presenting between 1^st^ August 2010 and 31^st^ December 2014.The biodata, pre and post-transplant details of these patients were documented. The data was analysed using SPSS Vs 17.

**Results:**

A total of 47 patients were studied with M: F ratio of 4:1, the mean age was 45.4 ± 13.6 years. Chronic glomerulonephritis, hypertension, diabetes mellitus and HIV related kidney disease were the commonest cause of CKD. Financial constraint delayed transplant in 66% and non-availability of donor in 17.2%. About 90% of the transplants were in India and 81% either financed the transplant either directly or through a relation. There was no cadaveric transplant and about 70% of the donors were not related. Tacrolimus, mycophenolate and prednisolone were most frequently used immunosuppressive combination. The one and three years graft survival were 95.3% and 67.6% respectively while corresponding patients survival were 97.7% and 82.4% respectively. Septicaemia, acute rejection and urinary tract infection were most common complications.

**Conclusion:**

Management of patients with kidney transplant has good prospect despite the challenges.

## Introduction

Human organ transplantation started in the twentieth century, however surgical transplantation of human organs from deceased, as well as living donors to sick and dying patients began after the Second World War [1]. Marked improvements in early graft survival and long-term graft function have made kidney transplantation a more cost-effective alternative to dialysis. Thus renal transplantation has become the treatment of choice for most patients with end-stage renal disease (ESRD). However barriers to universal transplantation as the therapy for end-stage kidney disease are many and include the economic limitations which, in resource poor countries, place transplantation, at a lower priority than public health fundamentals such as clean water, sanitation and vaccination. Even in high-income countries, the technical challenges of surgery and the consequences of immunosuppression restrict the number of suitable recipients, but the major finite restrictions on kidney transplantation rates are the shortage of donated organs and the limited medical, surgical and nursing work forces with the required expertise. Since the first successful kidney transplant between identical twins performed in Boston on 23^rd^ Dec 1954 [2], there has been remarkable advancement in the knowledge and technicalities of pre, intra and post-operative management of kidney transplant. This has impacted positively on both the short and long term survival of patients with kidney transplant. However there is remarkable disparity in the access and outcome of kidney transplant across the globe. Multiple immunologic and non-immunologic factors contribute to social, cultural and economic disparities in transplant outcomes, including biological, immune, genetic, metabolic, and pharmacological factors as well as associated comorbidities, time on dialysis, donor and organ characteristics, patient socio-economic status, medication adherence, access to care, and public health policies [3]. Developing countries often have especially poor transplant rates not only because of these multiple interacting factors, but also because of inferior infrastructure and an insufficiently trained workforce. In Nigeria with a population of about 170 million [4], the management of Kidney diseases including ESRD is still evolving. There are about 150 nephrologists, 120 dialysis centers (largely owned and manned by non-nephrologist), fewer trained dialysis nurses and technicians. Kidney transplantation including pre and post-transplant care are rudimentary. Prior to the first kidney transplant done within the country about a decade and half ago in a private kidney care centre, few kidney transplants recorded in Nigeria then were done in the United Kingdom. Up to date about 200 live related kidney transplants have been carried out in 7 transplant Centre’s in Nigeria [5]. However this represents less than 10% of Nigerians that had kidney transplant. This presents with antecedent challenges of managing these patients with background of profound limitations in resources, personnel and equipment. There is only a study on kidney transplant in Nigeria, and the study focused mainly on patients who were transplanted locally. The outcome in that study was poorer than those in developed countries. All the donors in that study were live related and the challenges of adherence to medications were highlighted. There is a need for a study that will include all the patients who had kidney transplant irrespective of the country of transplant. It will highlight the challenges of managing patients with kidney transplant including the limitations that the care givers including nephrologist encounter with these patients. The outcome of the transplant will be more representative of kidney transplant patients in Nigeria because majority of the transplant patients in Nigeria had their transplant abroad. The objective of this study is to evaluate the management of patients with kidney transplant in Nigeria using a kidney care centre in Nigeria as surrogate.

## Methods

This is a non-randomized prospective study. The study location is Hilton clinics, a 25 bedded private hospital located in the Southern region of Nigeria. The centre attends to patients with various types and severities of kidney and kidney related diseases. It offers various diagnostic and therapeutic renal procedures including renal imaging, renal biopsy, hemodialysis, vascular access creation, pre transplant work up and post-transplant care. The study populations were post kidney transplant patients that attended the centre between 1^st^ August 2010 and 31^st^ December 2014. The details of the study were explained to the patients and informed consent was obtained from each of them before proceeding to the study. The study was approved by the ethical committee of the institution. The demographic characteristics, clinical details of the patients and the transplant details including the indication for the kidney transplant, cause of the kidney disease, type of donor, country of the kidney transplant, sponsor of the transplant, type of immunosuppression, complication and outcome of the kidney transplant were documented. These information were obtained from the patient, patient’s relatives, the transplant and post-transplant follow up notes.

### Statistical analysis

The data obtained were entered in a spread sheet and analysed using statistical package for the social sciences (SPSS) version 17(IBM Inc.2010). Categorical data are reported as frequencies and percentages, and continuous variables are reported as mean and standard deviation. The patient and graft survival were calculated using Kaplan Meier estimator.

## Results

### Biodata

Forty seven patients that presented during the study period were enrolled for this study. Males constitute 76.6% (36) with M: F ratio of 4:1. The age range of the patients was 24 to 65 years with mean of 45.4±13.6 years. Nineteen (40%) were civil servants, 27.7% were unemployed and were either students, housewives or retiree.

### Kidney disease

**Figure 1:**
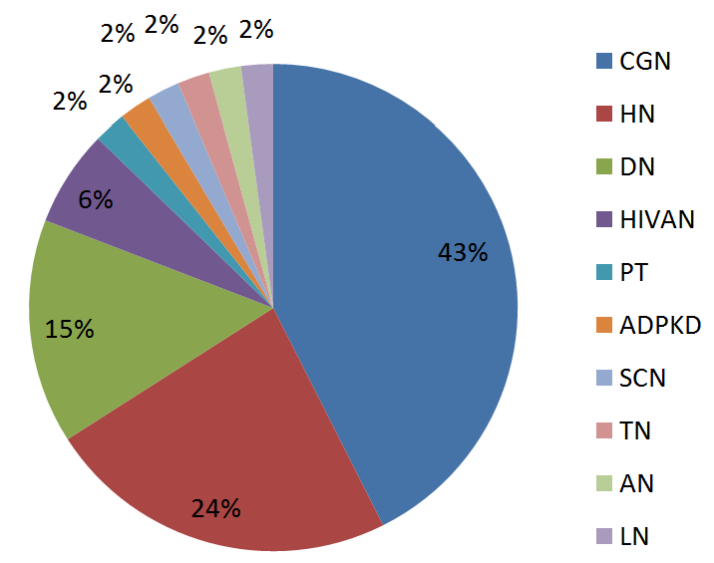
Distribution of kidney diseases in the transplant patients

The distribution of the causes of kidney disease in these patients is as documented in Figure 1. All the patients were in end stage kidney disease (ESRD), all the patients except one were on maintenance haemodialysis before transplant. None of the patient was on peritoneal dialysis.

### Kidney transplant

**Figure 2:**
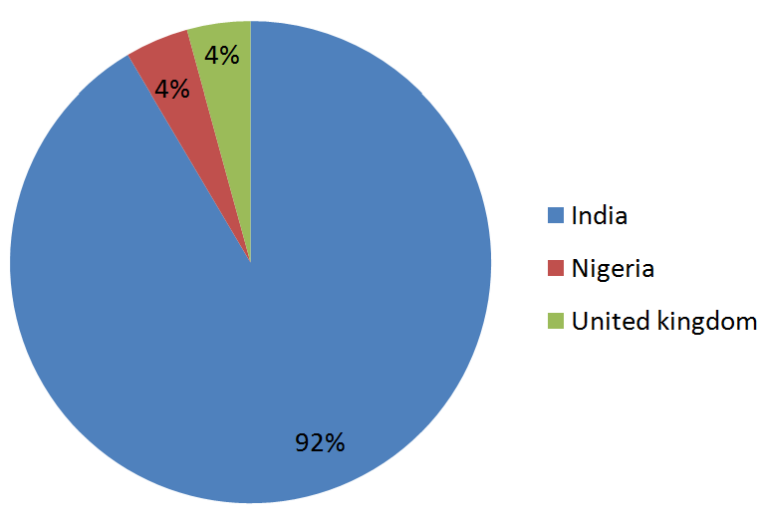
Country of transplant

The major constraint leading to delay of the transplant was finance, and this was found in 66% of the patients. Other factors that delayed transplant were availability of donor (17.2%) and medical condition (4.3%). However only 12.8% (6) patients had the transplant unhindered including one that was transplanted after few salvage haemodialysis. The location of the transplant centre is shown in Figure 2. The kidney transplant was financed by the patient in 42.6%, family in 36.2%, employer in 8.5%, church in 4.3%, friends in 2.2% and government in 2.2%. All the donors were living, however only 25.5% were related biologically, 4.3% were emotionally related (spouse) and 70.2% were neither biologically nor emotionally related. Less than half (42.5%) of the patients were followed up by a local nephrologist prior to development of complication including graft failure, 48.9% were followed up via telemedicine by the nephrologist from the transplant centre abroad and about 8.5% were not followed up.

### Immunosuppression

**Figure 3:**
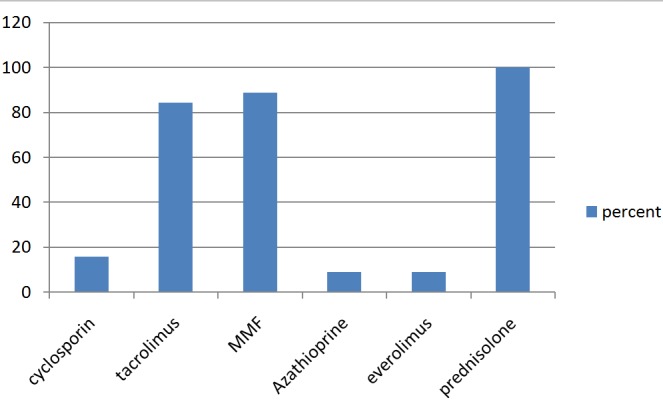
Immunosuppressive drug

Thirty eight (80.1%) patients received pretransplant and intraoperative induction imunosuppresive therapy. All the patients were initially on triple regimen of calnineurin inhibitor (cyclosporine or tacrolimus), steroid (prednisolone) and azathioprione or mycophenolate for maintenance immunosuppression. However following complication, everolimus was added in 2 patients, replaced tacrolimus in 1 patient and mycophenolate in 1 patient. Forty two (89.4%) procured their medication from India. The blood calcineurin inhibitor level was assessed regularly in only 19.1% of patients. Details of the immunosppressive drug is shown in Figure 3.

### Outcome

**Figure 4:**
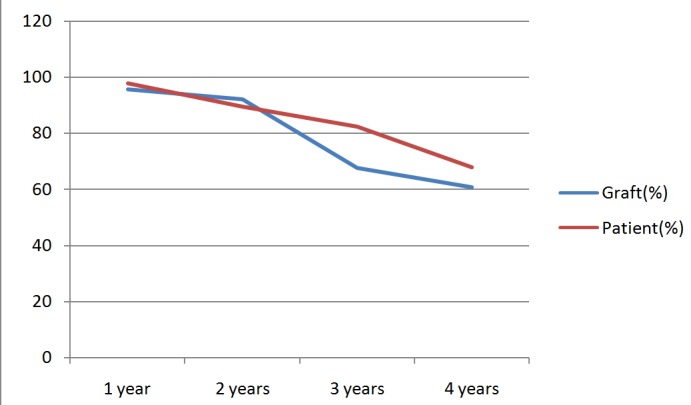
Graft and patient survival

The immediate outcome was good in 68.1%, fair in 29.8% and poor in 2.2%. 29.8% of the patients died during the study. The 1 to 4 years graft and patient survival is documented in Figure 4. One year graft survival was 95.3%, 2 years survival was 92.1%, 3 years survival was 67.6%, 4 years survival 60.7%. The patient’s survival was 1 year 97.7%, 2 years 89.5%, 3 years 82.4%, 4 years 67.9%.

### Complications

The common complications include urinary tract infection, septicaemia and acute rejection. The details of the complication are documented in Table 1.

**Table 1:** Distribution of complication

Complications	Frequency	Percent
Acute rejection	14	29.8
Septicaemia	13	27.6
Urinary tract infection	5	10.6
Peptic ulcer disease	2	4.3
Stroke	2	4.3
Congestive cardiac failure	2	4.3
Haematuria	2	4.3
Delayed graft function	2	4.3
Myocardiac infarction	2	4.3
Chronic allograft nephropathy	2	4.3
Kaposi saecoma	1	2.1
Gingivial hypertrophy	1	2.1
Hepatitis B infection	1	2.1
Pulmonary tuberculosis	1	2.1
Diabetes mellitus	1	2.1

## Discussion

The prevalence of chronic kidney disease (CKD) and end stage kidney disease (ESRD) in Nigeria is enormous affecting over 10% of the population and constituting about 8% of medical admissions [6–8]. The distribution of the cause of ESRD in this study was consistent with previous community and hospital based studies on causes of CKD in Nigeria. The young age group, the low-middle socio economic class and the males are more prevalent in this study as CKD is more prevalent in this group in Nigeria [6, 8]. However the males are more likely to have kidney transplant as depicted by the high M: F ratio of 4:1. This could be attributed to various sociocultural peculiarities of the Nigerian population where the male sex is preferred. Kidney transplant is the most appropriate renal replacement therapy (RRT) for patients with ESRD in terms of survival and quality of life. Preemptive transplant or transplant as primary RRT is encouraged in populations like Nigeria where other modalities of RRT are unaffordable, unsustainable and unavailable. However all the patients in this study had ESRD and were on maintenance haemodialysis preceding the kidney transplant except a patient who had kidney transplant as primary RRT. This is consistent with report of only 2.5% of incident patients with end-stage renal disease undergoing transplantation as their initial modality of treatment [9]. It is noteworthy that despite the various advances in kidney transplant this figure has remained largely unchanged for at least a decade. Poverty [10] is a major limitation to obtaining kidney transplant in most resource poor and developing countries including Nigeria. In this study financial constraint caused delay in procuring kidney transplant in 66% of patients while non-availability of kidney donor as a hindrance to kidney transplant was found in 17.2%. This is in contrast to reports from most developed countries where the major determinant of timing of kidney transplant was availability of donor [11]. Kidney transplant has been reported in less than 5% of patients in Nigerians requiring kidney transplant [5] which is very low when compared with similar reports from other countries especially the developed countries [12]. Factors responsible for this low prevalence of kidney transplant in Nigeria and most developing/disadvantaged population has been adduced in addition to poverty to include few/non availability of transplant facility, ignorance, lack of transplant and transplant related policies, lack of subsidy or rebate in funding of kidney transplant, lack of laboratory and pharmaceutical support. More than 95% of the patients in this study had their transplant abroad with 92% of them in India, also drug level assessment and most medications are sourced from overseas. Funding from health insurance and support from either governmental or nongovernmental organizations are insignificant or nonexistent, thus leaving the funding of the transplant and its management as documented in this study to patients and their relatives. The donors in this study were only living donors as patients were not qualified or there is no facility for cadaveric transplantation. Follow up of these patients was skewed, as about a 60% of the patients were either not followed up or depend on follow up through telemedicine by the nephrologist at the transplant centre abroad. It is not surprising that these patients had poorer outcome. This malady of dependence of overseas treatment is not limited to kidney transplant as there has been report of rampant medical tourism and overdependence in medical facilities in some Asian and western countries by Nigerians. Lack of health and transplant friendly government policies has perpetuated this poor state of kidney transplant activity in the country. Recently a national health act was enacted in Nigeria, it is hoped that with appropriate implementation it will impact positively on the management of patients including those requiring kidney transplant in the country. Sustenance of the functions of the transplanted kidney and survival of the transplanted patient depends largely on appropriate immunosuppression. About 80% of the patients in this study received induction immunosuppression probably because they were blacks and also majority of the donors were not related. The triple regimen of tacrolimus, mycophenolate and prednisolone was the immunosuppressive used in about 90% of the patients. It was noted that all these patients were transplanted in India as cyclosporine was the calcineurin inhibitor in patients transplanted elsewhere. Everolimus was used as intervention drugs either as a substitute or add on drug following development of complications in these patients. The blood level of calcineurin inhibitors were not monitored in most of the patients as prescribed by the K – DOQI guideline and this is due to financial constraint. It is also of note that the facility for the laboratory assessment of the calcineurin blood level is sparsely available in Nigeria. Almost all the samples for calcineurin level were taken abroad for analysis, resulting in over a week delay before obtaining the results. However some diagnostic centres are emerging with expectation of alleviating this hindrance in management of patients with kidney transplant in Nigeria. The one and two years patients and graft survival in this study is comparable to reports from both developed and developing countries [5, 13, 14]. However the 3 and 4 years survival were poorer than reports from countries with better developed transplant policies and facilities but comparable to reports from resource poor nations. About 30% of the patients died during the study period (4 years) with septicaemia incriminated in about 50% of them. Outcome of kidney transplant in this study is much better than abysmal outcomes reported in other modalities of RRT in Nigeria especially haemodialysis which is the most prevalent RRT [15]. This implies that kidney transplant should be recommended as primary RRT and patients counseled on need of early kidney transplant. There should be advocacy and interventions to improve kidney transplant including funding and widespread enlightenment to both the populace and policy makers. Acute rejection, infections and cardiovascular complications are the commonest complications documented in this study. The patient with gingival hypertrophy improved on withdrawal of the antihypertensive amlodipine, Kaposi sarcoma resolved on commencement of everolimus, and new onset diabetes was noted to be steroid induced as it improved remarkably when steroid dose was reduced. These complications have been documented in other studies [5, 16].

## Conclusion

In conclusion, management of patients with kidney transplant in Nigeria is froth with many financial, intellectual and infrastructural challenges however the outcome is relatively good and comparatively better than other existing RRT. The prospect of kidney transplant in Nigeria is good. Thus there is need for consented effort by the government, nongovernmental organizations and industries to invest in the development of kidney transplant in Nigeria.

### What is known about this topic

- Chronic kidney disease is prevalent;
- Kidney transplant centres are few in Nigeria;
- Kidney transplant is expensive.

### What this study adds

- Majority of kidney transplant patients in Nigeria had their transplant in India;
- Unrelated live donation is common;
- Outcome is relatively good.
